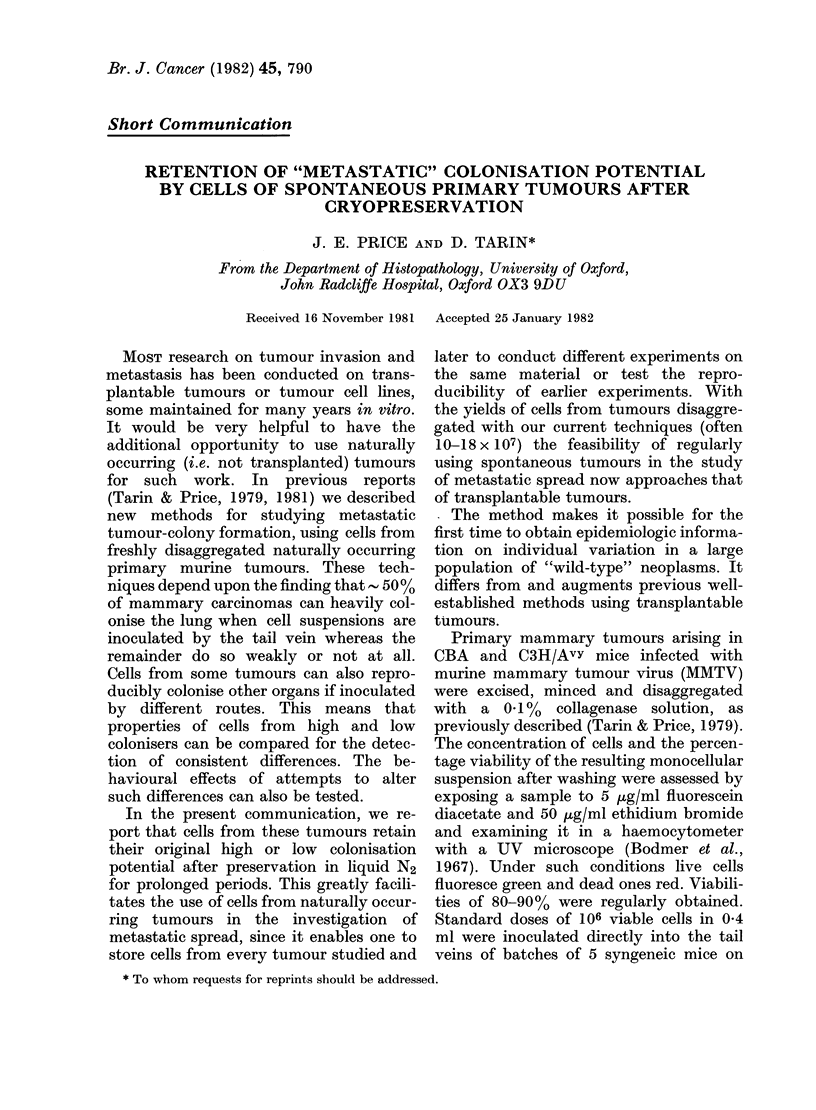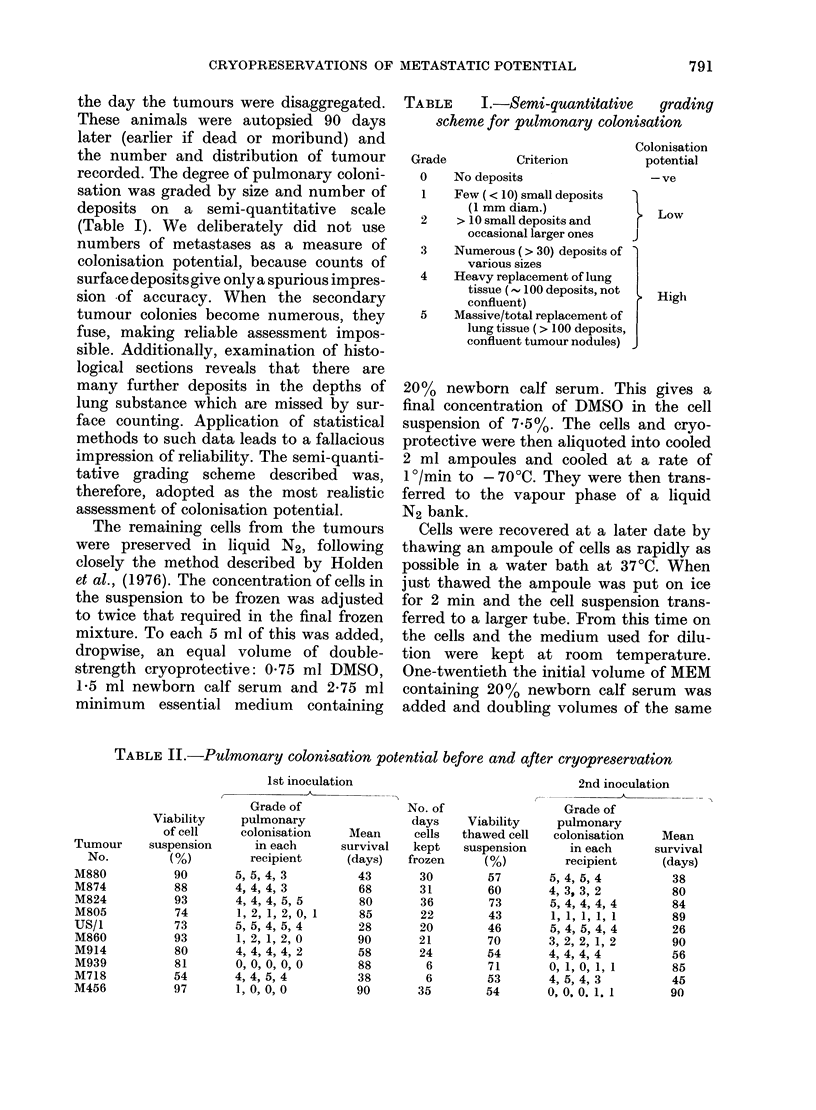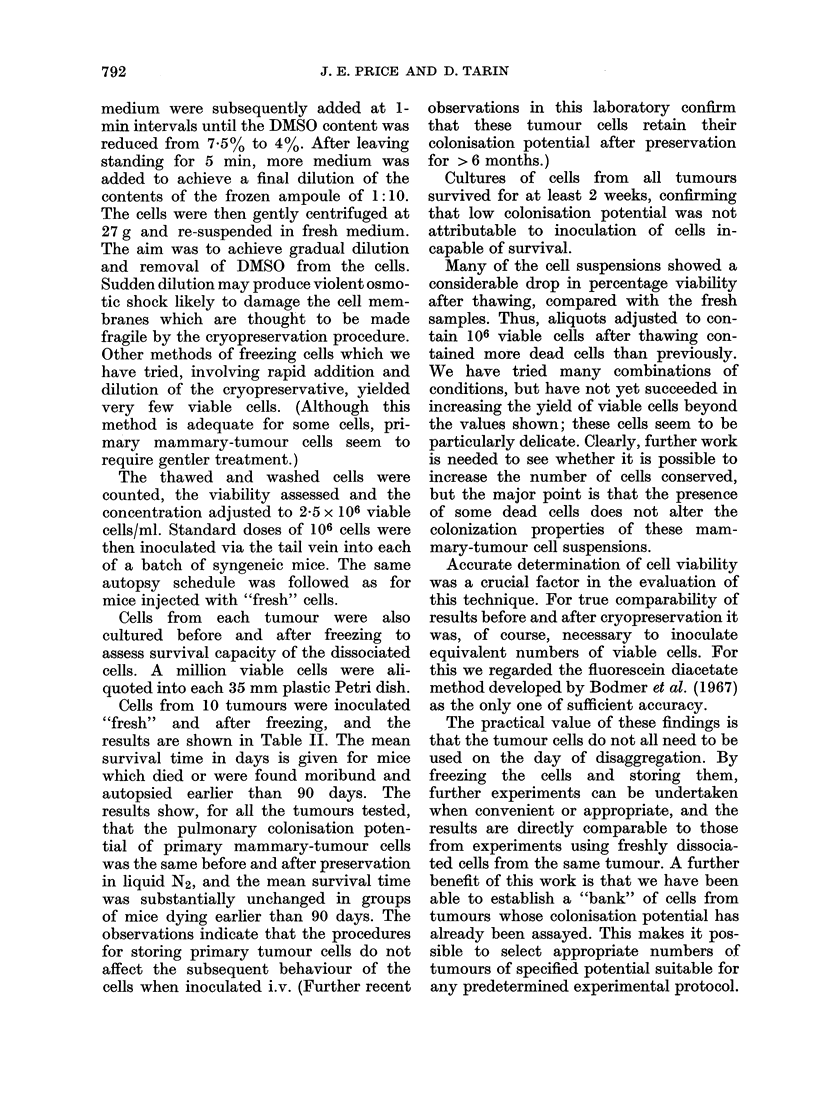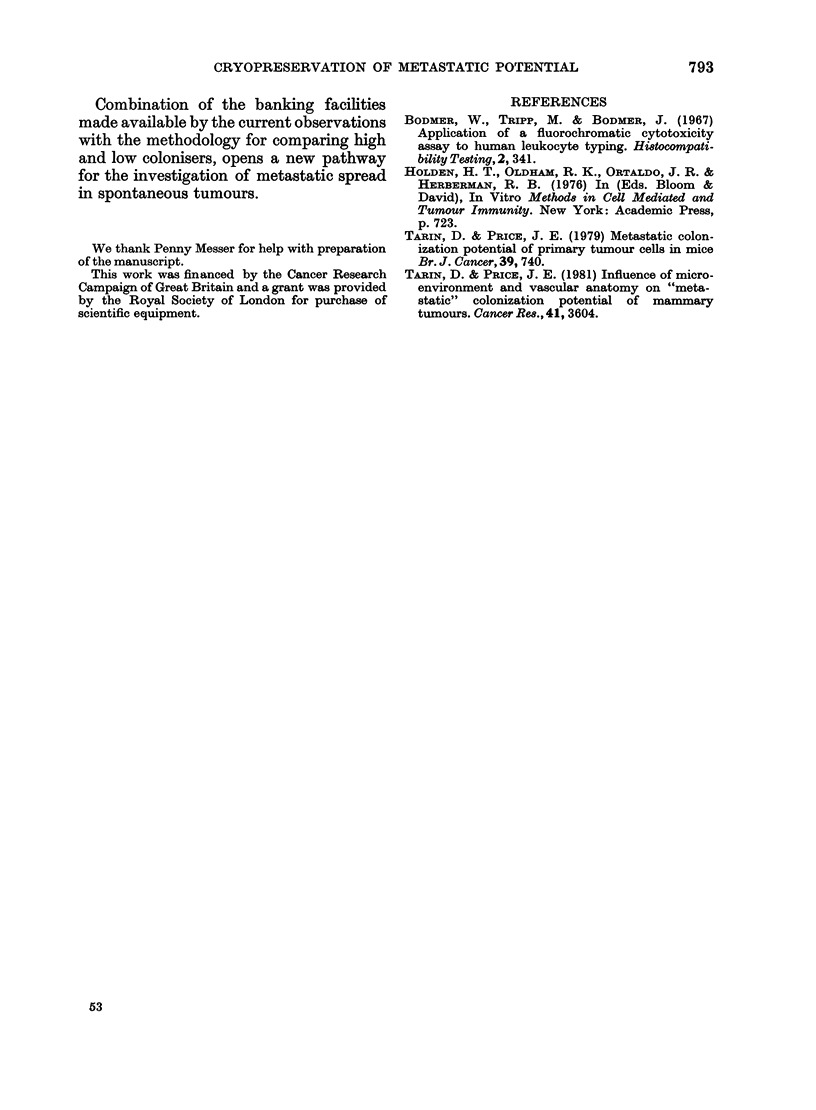# Retention of "metastatic" colonisation potential by cells of spontaneous primary tumours after cryopreservation.

**DOI:** 10.1038/bjc.1982.123

**Published:** 1982-05

**Authors:** J. E. Price, D. Tarin


Br. J. Cancer (1982) 45, 790

*Short Communication*

### RETENTION OF "METASTATIC" COLONISATION POTENTIAL BY CELLS OF SPONTANEOUS PRIMARY TUMOURS AFTER CRYOPRESERVATION

J. E. PRICE AND D. TARIN*

*From the Department of Histopathology, University of Oxford, John Radcliffe Hospital, Oxford OX3 9DU*

Received 16 November 1981 Accepted 25 January 1982

MOST research on tumour invasion and metastasis has been conducted on transplantable tumours or tumour cell lines, some maintained for many years *in vitro*. It would be very helpful to have the additional opportunity to use naturally occurring (*i.e.* not transplanted) tumours for such work. In previous reports (Tarin & Price, 1979, 1981) we described new methods for studying metastatic tumour-colony formation, using cells from freshly disaggregated naturally occurring primary murine tumours. These techniques depend upon the finding that ~ 50% of mammary carcinomas can heavily colonise the lung when cell suspensions are inoculated by the tail vein whereas the remainder do so weakly or not at all. Cells from some tumours can also reproducibly colonise other organs if inoculated by different routes. This means that properties of cells from high and low colonisers can be compared for the detection of consistent differences. The behavioural effects of attempts to alter such differences can also be tested.

In the present communication, we report that cells from these tumours retain their original high or low colonisation potential after preservation in liquid $N_2$ for prolonged periods. This greatly facilitates the use of cells from naturally occurring tumours in the investigation of metastatic spread, since it enables one to store cells from every tumour studied and later to conduct different experiments on the same material or test the reproducibility of earlier experiments. With the yields of cells from tumours disaggregated with our current techniques (often $10–18 \times 10^7$) the feasibility of regularly using spontaneous tumours in the study of metastatic spread now approaches that of transplantable tumours.

The method makes it possible for the first time to obtain epidemiologic information on individual variation in a large population of "wild-type" neoplasms. It differs from and augments previous well-established methods using transplantable tumours.

Primary mammary tumours arising in CBA and C3H/$A^{vy}$ mice infected with murine mammary tumour virus (MMTV) were excised, minced and disaggregated with a 0·1% collagenase solution, as previously described (Tarin & Price, 1979). The concentration of cells and the percentage viability of the resulting monocellular suspension after washing were assessed by exposing a sample to 5 $\mu$g/ml fluorescein diacetate and 50 $\mu$g/ml ethidium bromide and examining it in a haemocytometer with a UV microscope (Bodmer *et al.*, 1967). Under such conditions live cells fluoresce green and dead ones red. Viabilities of 80–90% were regularly obtained. Standard doses of $10^6$ viable cells in 0·4 ml were inoculated directly into the tail veins of batches of 5 syngeneic mice on

* To whom requests for reprints should be addressed.

the day the tumours were disaggregated. These animals were autopsied 90 days later (earlier if dead or moribund) and the number and distribution of tumour recorded. The degree of pulmonary colonisation was graded by size and number of deposits on a semi-quantitative scale (Table I). We deliberately did not use numbers of metastases as a measure of colonisation potential, because counts of surface deposits give only a spurious impression of accuracy. When the secondary tumour colonies become numerous, they fuse, making reliable assessment impossible. Additionally, examination of histological sections reveals that there are many further deposits in the depths of lung substance which are missed by surface counting. Application of statistical methods to such data leads to a fallacious impression of reliability. The semi-quantitative grading scheme described was, therefore, adopted as the most realistic assessment of colonisation potential.

The remaining cells from the tumours were preserved in liquid $N_2$, following closely the method described by Holden *et al.*, (1976). The concentration of cells in the suspension to be frozen was adjusted to twice that required in the final frozen mixture. To each 5 ml of this was added, dropwise, an equal volume of double-strength cryoprotective: 0·75 ml DMSO, 1·5 ml newborn calf serum and 2·75 ml minimum essential medium containing

TABLE I.—*Semi-quantitative grading scheme for pulmonary colonisation*

| Grade | Criterion | Colonisation potential |
|---|---|---|
| 0 | No deposits | −ve |
| 1 | Few (< 10) small deposits (1 mm diam.) | Low |
| 2 | > 10 small deposits and occasional larger ones | Low |
| 3 | Numerous (> 30) deposits of various sizes | High |
| 4 | Heavy replacement of lung tissue (~ 100 deposits, not confluent) | High |
| 5 | Massive/total replacement of lung tissue (> 100 deposits, confluent tumour nodules) | High |

20% newborn calf serum. This gives a final concentration of DMSO in the cell suspension of 7·5%. The cells and cryoprotective were then aliquoted into cooled 2 ml ampoules and cooled at a rate of 1°/min to −70°C. They were then transferred to the vapour phase of a liquid $N_2$ bank.

Cells were recovered at a later date by thawing an ampoule of cells as rapidly as possible in a water bath at 37°C. When just thawed the ampoule was put on ice for 2 min and the cell suspension transferred to a larger tube. From this time on the cells and the medium used for dilution were kept at room temperature. One-twentieth the initial volume of MEM containing 20% newborn calf serum was added and doubling volumes of the same

TABLE II.—*Pulmonary colonisation potential before and after cryopreservation*

| | 1st inoculation | | | | 2nd inoculation | | |
|---|---|---|---|---|---|---|---|
| Tumour No. | Viability of cell suspension (%) | Grade of pulmonary colonisation in each recipient | Mean survival (days) | No. of days cells kept frozen | Viability thawed cell suspension (%) | Grade of pulmonary colonisation in each recipient | Mean survival (days) |
| M880 | 90 | 5, 5, 4, 3 | 43 | 30 | 57 | 5, 4, 5, 4 | 38 |
| M874 | 88 | 4, 4, 4, 3 | 68 | 31 | 60 | 4, 3, 3, 2 | 80 |
| M824 | 93 | 4, 4, 4, 5, 5 | 80 | 36 | 73 | 5, 4, 4, 4, 4 | 84 |
| M805 | 74 | 1, 2, 1, 2, 0, 1 | 85 | 22 | 43 | 1, 1, 1, 1, 1 | 89 |
| US/1 | 73 | 5, 5, 4, 5, 4 | 28 | 20 | 46 | 5, 4, 5, 4, 4 | 26 |
| M860 | 93 | 1, 2, 1, 2, 0 | 90 | 21 | 70 | 3, 2, 2, 1, 2 | 90 |
| M914 | 80 | 4, 4, 4, 4, 2 | 58 | 24 | 54 | 4, 4, 4, 4 | 56 |
| M939 | 81 | 0, 0, 0, 0, 0 | 88 | 6 | 71 | 0, 1, 0, 1, 1 | 85 |
| M718 | 54 | 4, 4, 5, 4 | 38 | 6 | 53 | 4, 5, 4, 3 | 45 |
| M456 | 97 | 1, 0, 0, 0 | 90 | 35 | 54 | 0, 0, 0, 1, 1 | 90 |

medium were subsequently added at 1-min intervals until the DMSO content was reduced from 7·5% to 4%. After leaving standing for 5 min, more medium was added to achieve a final dilution of the contents of the frozen ampoule of 1:10. The cells were then gently centrifuged at 27 g and re-suspended in fresh medium. The aim was to achieve gradual dilution and removal of DMSO from the cells. Sudden dilution may produce violent osmotic shock likely to damage the cell membranes which are thought to be made fragile by the cryopreservation procedure. Other methods of freezing cells which we have tried, involving rapid addition and dilution of the cryopreservative, yielded very few viable cells. (Although this method is adequate for some cells, primary mammary-tumour cells seem to require gentler treatment.)

The thawed and washed cells were counted, the viability assessed and the concentration adjusted to $2 \cdot 5 \times 10^6$ viable cells/ml. Standard doses of $10^6$ cells were then inoculated via the tail vein into each of a batch of syngeneic mice. The same autopsy schedule was followed as for mice injected with "fresh" cells.

Cells from each tumour were also cultured before and after freezing to assess survival capacity of the dissociated cells. A million viable cells were aliquoted into each 35 mm plastic Petri dish.

Cells from 10 tumours were inoculated "fresh" and after freezing, and the results are shown in Table II. The mean survival time in days is given for mice which died or were found moribund and autopsied earlier than 90 days. The results show, for all the tumours tested, that the pulmonary colonisation potential of primary mammary-tumour cells was the same before and after preservation in liquid $N_2$, and the mean survival time was substantially unchanged in groups of mice dying earlier than 90 days. The observations indicate that the procedures for storing primary tumour cells do not affect the subsequent behaviour of the cells when inoculated i.v. (Further recent observations in this laboratory confirm that these tumour cells retain their colonisation potential after preservation for > 6 months.)

Cultures of cells from all tumours survived for at least 2 weeks, confirming that low colonisation potential was not attributable to inoculation of cells incapable of survival.

Many of the cell suspensions showed a considerable drop in percentage viability after thawing, compared with the fresh samples. Thus, aliquots adjusted to contain $10^6$ viable cells after thawing contained more dead cells than previously. We have tried many combinations of conditions, but have not yet succeeded in increasing the yield of viable cells beyond the values shown; these cells seem to be particularly delicate. Clearly, further work is needed to see whether it is possible to increase the number of cells conserved, but the major point is that the presence of some dead cells does not alter the colonization properties of these mammary-tumour cell suspensions.

Accurate determination of cell viability was a crucial factor in the evaluation of this technique. For true comparability of results before and after cryopreservation it was, of course, necessary to inoculate equivalent numbers of viable cells. For this we regarded the fluorescein diacetate method developed by Bodmer *et al.* (1967) as the only one of sufficient accuracy.

The practical value of these findings is that the tumour cells do not all need to be used on the day of disaggregation. By freezing the cells and storing them, further experiments can be undertaken when convenient or appropriate, and the results are directly comparable to those from experiments using freshly dissociated cells from the same tumour. A further benefit of this work is that we have been able to establish a "bank" of cells from tumours whose colonisation potential has already been assayed. This makes it possible to select appropriate numbers of tumours of specified potential suitable for any predetermined experimental protocol.

Combination of the banking facilities made available by the current observations with the methodology for comparing high and low colonisers, opens a new pathway for the investigation of metastatic spread in spontaneous tumours.

We thank Penny Messer for help with preparation of the manuscript.

This work was financed by the Cancer Research Campaign of Great Britain and a grant was provided by the Royal Society of London for purchase of scientific equipment.

### REFERENCES

BODMER, W., TRIPP, M. & BODMER, J. (1967) Application of a fluorochromatic cytotoxicity assay to human leukocyte typing. *Histocompatibility Testing*, **2**, 341.

HOLDEN, H. T., OLDHAM, R. K., ORTALDO, J. R. & HERBERMAN, R. B. (1976) In (Eds. Bloom & David), In Vitro *Methods in Cell Mediated and Tumour Immunity*. New York: Academic Press, p. 723.

TARIN, D. & PRICE, J. E. (1979) Metastatic colonization potential of primary tumour cells in mice *Br. J. Cancer*, **39**, 740.

TARIN, D. & PRICE, J. E. (1981) Influence of microenvironment and vascular anatomy on "metastatic" colonization potential of mammary tumours. *Cancer Res.*, **41**, 3604.